# Droplet Digital PCR Detection of the Erythropoietin Transgene from Horse Plasma and Urine for Gene-Doping Control

**DOI:** 10.3390/genes10030243

**Published:** 2019-03-21

**Authors:** Teruaki Tozaki, Aoi Ohnuma, Masaki Takasu, Mio Kikuchi, Hironaga Kakoi, Kei-ichi Hirota, Kanichi Kusano, Shun-ichi Nagata

**Affiliations:** 1 Genetic Analysis Department, Laboratory of Racing Chemistry, 1731-2 Tsurutamachi, Utsunomiya, Tochigi 320-0851, Japan; a-ohnuma@lrc.or.jp (A.O.); m-kikuchi@lrc.or.jp (M.K.); h-kakoi@lrc.or.jp (H.K.); k-hirota@lrc.or.jp (K.-i.H.); s-nagata@lrc.or.jp (S.-i.N.); 2 Department of Veterinary Medicine, Faculty of Applied Biological Sciences, Gifu University, 1-1 Yanagido, Gifu, Gifu 501-1193, Japan; takasu@gifu-u.ac.jp; 3 Racehorse Hospital Ritto Training Center, Japan Racing Association, 1028 Misono, Ritto, Shiga 520-3085, Japan; Kanichi_Kusano@jra.go.jp

**Keywords:** genome-to-phenome, exercise traits, morphologic traits, equine genetics, domestic horse, disease mechanisms

## Abstract

Indiscriminate genetic manipulation to improve athletic ability is a major threat to human sports and the horseracing industry, in which methods involving gene-doping, such as transgenesis, should be prohibited to ensure fairness. Therefore, development of methods to detect indiscriminate genetic manipulation are urgently needed. Here, we developed a highly sensitive method to detect horse erythropoietin (*EPO*) transgenes using droplet digital PCR (ddPCR). We designed two TaqMan probe/primer sets, and the *EPO* transgene was cloned into a plasmid for use as a model. We extracted the spiked *EPO* transgene from horse plasma and urine via magnetic beads, followed by ddPCR amplification for absolute quantification and transgene detection. The results indicated high recovery rates (at least ~60% and ~40% in plasma and urine, respectively), suggesting successful detection of the spiked transgene at concentrations of >130 and 200 copies/mL of plasma and urine, respectively. Additionally, successful detection was achieved following intramuscular injection of 20 mg of the *EPO* transgene. This represents the first study demonstrating a method for detecting the *EPO* transgene in horse plasma and urine, with our results demonstrating its efficacy for promoting the control of gene-doping in the horseracing industry.

## 1. Introduction

Horseracing began in Britain in the early 18th century and currently occurs worldwide. It is necessary for the sport to be conducted fairly under common rules; therefore, doping control is a primary concern for horseracing authorities [1], as it is important to ensure both fairness in the race and the health and welfare of the racehorses.

Prohibited substances identified for doping control include low-molecular-weight compounds, such as β-agonists and steroids [2,3]. However, recent developments in veterinary medicine have resulted in genetic doping (i.e., the illegal use of gene therapy) becoming a concern in the horseracing industry [4]. In 2017, the International Federation of Horseracing Authority revised their regulations on gene therapy used in racehorses for the purpose of gene-doping control (http://www.horseracingintfed.com), with “administration of oligomers and polymers of nucleic acids and nucleic acid analogues” stipulated as gene therapy in racehorses.

The oligomers of nucleic acids and nucleic acid analogues in this definition refer to therapeutic oligonucleotides [5]. The detection of phosphothioated oligonucleotides is accomplished using mass spectrometry (MS) [6], and polymers of nucleic acids and nucleic acid analogues in this definition refer to transgenes [7,8]. In gene therapy, plasmids, adeno-associated viruses, adenoviruses, and other vehicles are used as vectors to introduce genes, with the administered transgenes supplementing the functions of defective genes. In gene therapy research involving horses, examples of transgene therapy include administering *bone morphogenetic protein 2* (*BMP2)* and *BMP6* to a model fracture site using an adenoviral vector [9] and *fibroblast growth factor 2* and *vascular endothelial growth factor 164* to sites of tendinitis using a plasmid vector [10] in order to promote lesion healing.

Erythropoietin (EPO)-related medicines are problematic for doping control in human sports, especially in marathons and road bicycle racing due to their hematopoietic function. Thoroughbred horseracing is also a running competition typically involving races from 1000 to 3000 m that require endurance; therefore, erythropoietin-related substances, including transgenes, should be prohibited.

In conventional doping tests, such as those targeting a low-molecular-weight compound, MS is used [11], whereas for gene-doping, MS would not be optimal for transgene detection, because the vector sequences are several kilobases long. Therefore, detection based on polymerase chain reaction (PCR) has been proposed [12]. PCR allows amplification of specific DNA segments and is used for genetic trait diagnosis and parentage testing [13,14], making it efficacious for the development of detection methods targeting gene-doping. Digital PCR was recently developed and used for quantitative evaluation [15], as it enables the absolute quantitation of target sequences in samples.

In our previous study, we successfully detected a transgene administered to a microminipig using droplet digital PCR (ddPCR) as a model case study [16]. In this study, we developed and validated a method to detect gene-doping substances using the horse *EPO* gene cloned in a plasmid and used as a doping substance.

## 2. Materials and Methods

### 2.1. Ethical Considerations

Animal experiments in this study were approved by the Committee for Animal Research and Welfare of the Equine Research Institute (ERI), Japan Racing Association (JRA), and were conducted at facilities at the ERI, JRA, Shimotsuke, Japan.

### 2.2. Animal Genomic DNA Preparation

Genomic DNA from donkey, human, monkey, dog, cat, bovine, goat, sheep, porcine, mini-pig, camel, llama, mouse, rabbit, and chicken were purchased from Zyagen (San Diego, CA, USA). The genomic DNA was diluted to 20 ng/µL in Milli-Q water (Millipore, Billerica, MA, USA).

Blood sampling from thoroughbred racehorses for method development and casework examples was performed in the ERI and Miho Training Center, JRA, by veterinarians while ensuring no stress to the horses. Horse blood was collected into BD Vacutainer spray coated K2EDTA tubes (Becton Dickinson, Franklin Lakes, NJ, USA) or heparin tubes (Becton Dickinson), and plasma was separated by centrifugation at 1500× *g* for 10 min and stored at −40 °C.

From these samples, DNA was extracted using a Prepito circulating NA 1k kit (Perkin Elmer, Waltham, MA, USA) and a Prepito-D (Perkin Elmer) instrument. The extract was dissolved with Milli-Q water (Millipore) at a final volume of 90 µL.

### 2.3. Preparation of Cloned Horse EPO as a Reference

Based on reference genomic information (EquCab2.0; GCA-000002305.1), we synthesized the 1180-bp horse EPO containing the open reading frame (ORF) and untranslated regions (Fasmac, Kanagawa, Japan). The synthesized sequence was then cloned into the plasmid vector pUCFa (r-Amp^+^, ColE1_ori^+^; p.horseEPO) and transfected into JM109 competent cells (Takara Bio, Shiga, Japan). The cells were cultured in Luria–Bertani medium (Amp^+^), followed by purification of p.horseEPO using a Wizard Plus SV miniprep DNA purification system (Promega, Madison, WI, USA) and solvation in Milli-Q water (Millipore). The sequence of the cloned p.horseEPO was confirmed by Sanger sequencing and subsequently used as reference material (RM) in this study.

### 2.4. Serial Dilution of p.horseEPO

Serially diluted (SD) samples were prepared from the RM by dilution using 10 ng/μL of horse genomic DNA (SD1.0 (1/10 of RM), SD2.0 (1/10 of SD1.0), SD3.0 (1/10 of SD2), SD4.0 (1/10 of SD3), SD4.5 (1/2 of SD4.0), SD5.0 (1/5 of SD4.5), SD5.5 (1/2 of SD5.0), SD6.0 (1/5 of SD5.5), SD6.5 (1/2 of SD6.0), SD7.0 (1/5 of SD6.5), SD7.5 (1/2 of SD7.0), SD8.0 (1/5 of SD7.5), SD8.5 (1/2 of SD8.0), SD9.0 (1/5 of SD8.5), and SD9.5 (1/2 of SD9.0)). SD samples were prepared for day 1 and day 2 experiments, respectively. For the preparation, 1.5 mL non-stick RNase-free microfuge tubes (Ambion; Thermo Fisher Scientific, Waltham, MA, USA) were used to avoid adsorption of p.horseEPO on the tube wall.

### 2.5. Evaluation of Purified p.horseEPO as a Reference Material

Reference material and SD samples were quantified by mass concentration (ng/μL) using a Qubit dsDNA HS assay kit (Thermo Fisher Scientific). Based on both the measured mass concentration (ng/μL) and molecular mass of p.horseEPO, we calculated the theoretical copy number concentration (copy/μL). To obtain the actual copy/μL of p.horseEPO, we used ddPCR. The relationship between concentrations was assessed by calculating the measured:theoretical ratio.

### 2.6. Sample Preparation for Spike-Recovery Tests

Samples for spike-recovery tests were prepared by adding 10 μL of serially diluted p.horseEPO (SD4.0–9.5) into 1 mL horse plasma (EDTA or heparin) or 1 mL horse urine. From these samples, p.horseEPO was extracted using a Prepito circulating NA 1k kit and a Prepito-D instrument. The extract was dissolved with Milli-Q water at a final volume of 90 µL.

### 2.7. Primer and Probe Design for ddPCR

To amplify the horse EPO transgene, we synthesized SET1 and SET2 probes (TaqMan–MGB) and primers (forward and reverse) (Table 1) (Thermo Fisher Scientific). Each primer (forward and reverse) was designed targeting different exons, and the probes were designed to target the junction of both exons. Sequence information for SET1 is withheld to support the integrity of gene-doping tests.

### 2.8. ddPCR Using a TaqMan Probe

For ddPCR using a TaqMan probe, we followed manufacturer instructions, which involved 1.1 μL or 8.8 μL of sample solution, 11 μL of 2× ddPCR Supermix (no dUTP), 0.2 μL of 100 μM forward primer, 0.2 μL of 100 μM reverse primer, and 0.6 μL of 10 μM TaqMan–MGB probe in a total volume of 22 μL. After creating a droplet with an automated droplet generator (Bio-Rad, Hercules, CA, USA), a 20-μL droplet for ddPCR was subsequently prepared. PCR was performed in a T100 thermal cycler (Bio-Rad) under the following conditions: enzyme activation at 95 °C for 10 min, followed by 40 cycles of denaturation at 94 °C for 30 s, and annealing/extension at 60 °C for 1 min. After enzyme deactivation for 10 min at 98 °C, the PCR products were stored at 12 °C. Samples were subsequently measured using a QX200 droplet reader (Bio-Rad). ddPCR was performed in triplicate, and the mean and standard deviation were calculated.

### 2.9. Preparation of Cloned Horse EPO

Based on the genomic information (EquCab2.0; GCA-000002305.1), we synthesized the 579-bp horse EPO containing the ORF (192 amino acids) and a termination codon (Fasmac). The synthesized sequence was then cloned into the plasmid vector pBApo-CMV (r-Amp^+^, CMV IE promoter, HSV TK poly A; Takara Bio.), and large-scale (20 mg) preparations of pBApo.horseEPO were performed by Takara Bio. Purified pBApo.horseEPO was filtered through a 0.22-μm filter to remove endotoxins, and the sequence was confirmed by Sanger sequencing.

### 2.10. Administration of pBApo.horseEPO

The minimum number of animals was used in this case study to comply with animal ethics and welfare guidelines. One thoroughbred horse (male, 11 years old, 465 kg) at the ERI, JRA, was intramuscularly injected with 20 mg of pBApo.horseEPO as the horse *EPO* transgene, followed by blood and urine collection.

Blood sampling into EDTA tubes and heparin tubes (Becton Dickinson) was performed at 15 min, 1 h, 3 h, 6 h, 12 h, 1 day, 2 days, 3 days, 4 days, 5 days, 6 days, 1 week, 2 weeks, 3 weeks, and 4 weeks after administration. After sampling, the collected blood was centrifuged, and plasma was separated and stored at −40 °C.

Urine sampling was performed at 50 min, 3 h, 6 h, 12 h, 1 day, 2 days, 3 days, 4 days, 5 days, 6 days, 1 week, 2 weeks, 3 weeks, and 4 weeks after administration. The volume and pH of the collected urine were measured prior to storage at −40 °C.

pBApo.horseEPO was extracted from 1 mL of plasma or urine using Chemagic Prepito reagent from the Prepito circulating NA 1K kit (Perkin Elmer).

## 3. Results and Discussion

### 3.1. ddPCR Quantitation and Limit of Detection (LOD)

**Figure 1:**
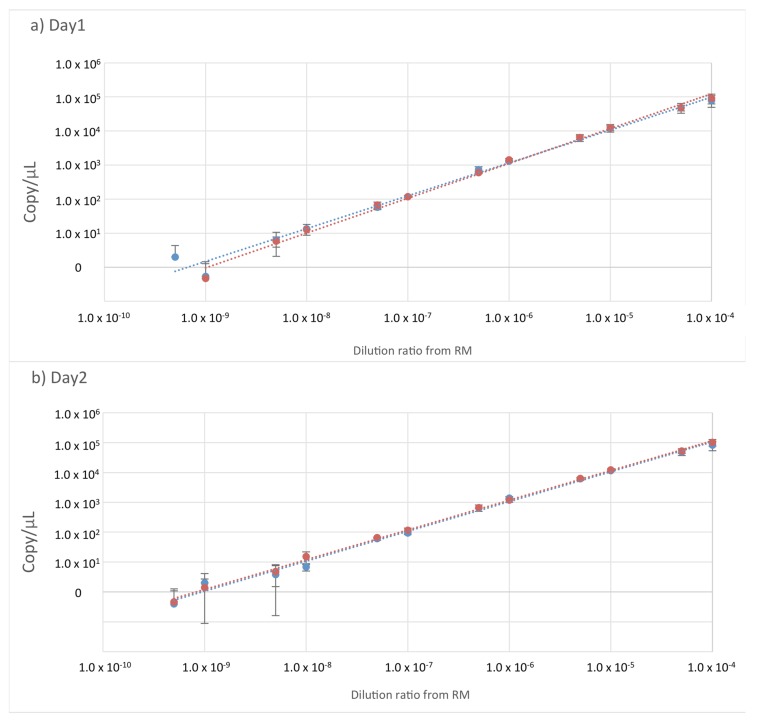
Droplet digital (dd)PCR quantitation and limit of detection (LOD). (**a**,**b**) Log–log graph with a regression line between serial dilutions and copy number concentrations at days 1 and 2, respectively. Blue circles denote use of SET1, and red circles denote SET2. The horizontal axis shows the dilution ratios from the reference material (RM) of serial dilution (SD)4.0 through SD9.5. The vertical axis shows copy number concentration in each SD. The slope indicates amplification efficiency.

The quantitative efficacy and LOD of ddPCR were validated using 1.1 μL of serially diluted p.horseEPO (SD4.0–9.5). The same experiment was performed twice (days 1 and 2) using the SET1 and SET2 primers and probes. Figure 1a,b show a log–log graph with a regression line between serial-dilution ratios and copy concentrations at days 1 and 2, respectively.

The correlation coefficients were 0.9983 and 0.9989 for SET1 and SET2 using SD4.0 through SD8.5 on day 1, with amplification efficiencies (slope) of 0.9676 and 0.9781, respectively (Figure 1a). At day 2, the correlation coefficients were 0.9959 and 0.9986 for SET1 and SET2 using SD4.0 through SD8.5, with amplification efficiencies of 1.0225 and 0.9889, respectively (Figure 1b). These results indicated that SET1 and SET2 were quantifiable at serial dilutions ranging from SD4.0 to SD8.5.

The copy numbers of SD8.5 in the ddPCR reaction were 6.3 ± 4.2 and 5.8 ± 1.9 at day 1 and 3.8 ± 3.6 and 4.8 ± 3.3 at day 2 for SET1 and SET2, respectively, whereas the mean copy number for SD9.0 and SD9.5 was <1.0 and showed a large standard deviation. These results indicated that, using SET1 and SET2, it was possible to detect at least seven copies in the ddPCR reaction (20 μL).

### 3.2. Reference Material Preparation and Evaluation

Concentrations of the RM and SD samples were measured as the mass concentration or copy number concentration (Table 2). Because the quantitative ranges of the mass concentration ranged from 0.2 ng/µL to 100 ng/μL, we only measured the RM in this study. Because the quantitative range of the copy number concentration using ddPCR according to the manufacturer was <100,000 copies, SD4.0 through SD9.5 were only quantified using SET1 and SET2. Theoretical copy number concentration was calculated from the mass concentration of the RM, and SD1.0 through SD9.5 were converted based on their dilution ratios.

To calculate a match rate between the measured and theoretical concentrations, we calculated the measured:theoretical ratio (Table 2). The ratios ranged from 0.384 to 0.825 for SET1 at days 1 and 2 using SD4.0 through SD8.5, respectively, and from 0.522 to 0.853 for SET2 at days 1 and 2 using SD4.0 through SD8.5, respectively. SD9.0 and SD9.5 did not show feasible ratios at the day 1 experiment. Overall, ddPCR results showed lower ratios relative to the concentrations converted from the mass concentration. For plasmid preparation, degraded RNA and DNA derived from *Escherichia coli* could not be completely removed, which might affect mass–concentration measurement.

As accurate quantification is required for RM preparation, ddPCR quantification is preferable as compared with mass–concentration measurement. For ddPCR quantification, SD4.0 though SD8.5 were suitable due to the measurement range. Additionally, dilution using a solution containing 10 ng/μL horse genomic DNA was also preferable to prevent wall adsorption. These procedures would be suitable steps for RM preparation and validation for gene-doping detection.

### 3.3. Evaluation of Anticoagulant in Collection Tubes

We performed a spike-recovery test to evaluate anticoagulant effect in blood-collection tubes. First, 10 μL of SD4 (high copy number) and SD6 (medium copy number) were added to 1 mL of horse plasma from EDTA and heparin collection tubes, followed by the extraction of spiked p.horseEPO with Prepito-D and quantification by ddPCR using primers and probes SET1 and SET2.

The recovery rates for SD4.0 and SD6.0 ranged from 66.9% to 99.2% when using plasma in EDTA collection tubes and 0% for at all samples when using plasma in heparin collection tubes. Because heparin is a known PCR inhibitor, residual heparin in the extracts likely influenced PCR amplification. These results indicated that plasma collected in EDTA collection tubes was preferable for transgene detection based on ddPCR.

### 3.4. PCR-Amplification Specificity

The specificity of the probes and primers (SET1 and SET2) designed to amplify the horse *EPO* transgene was confirmed using 20 ng of the human, monkey, dog, cat, bovine, goat, sheep, porcine, mini-pig, camel, llama, mouse, rabbit, chicken, donkey, and horse genomes as templates. ddPCR amplicons were not observed by the TaqMan method, whereas these amplicons were confirmed in some species using the intercalation method (data not shown). Although the primers were designed to target different exons, large-sized PCR products including the intron sequence might amplify, even when the intron sequence is short. Therefore, we determined that the TaqMan method was preferable for specific detection of transgenes without introns, and that detection of the horse *EPO* transgene was possible using the probes and primers designed in this study.

### 3.5. ddPCR Robustness

To evaluate ddPCR robustness, we tested different annealing/extension temperatures (52–65 °C) using the thermal gradient function in the T100 thermal cycler and 1.1 μL of SD4.0 as a template.

**Figure 2:**
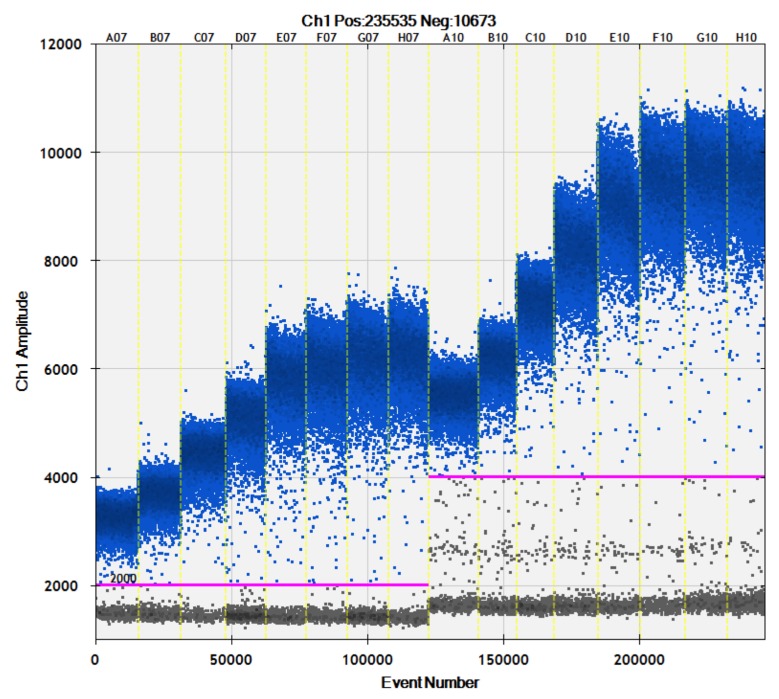
Robustness of ddPCR conditions for SET1 and SET2. The horizontal axis shows ddPCR amplifications using SD4.0 at different annealing/extension temperatures. Lanes 1 and 9: 65.0 °C, lanes 2 and 10: 64.0 °C, lanes 3 and 11: 62.4 °C, lanes 4 and 12: 59.9 °C, lanes 5 and 13: 57.0 °C, lanes 6 and 14: 54.4 °C, lanes 7 and 15: 52.9 °C, and lanes 8 and 16: 52.0 °C. Lanes 1 through 8 were amplified using SET1, and lanes 9 through 16 were amplified using SET2. The vertical axis shows PCR amplitudes (fluorescence intensity). Pink lines are detection thresholds.

At all annealing/extension temperatures, PCR products were well amplified using both SET1 and SET2. In particular, amplitudes at 57.0 °C, 54.4 °C, 52.9 °C, and 52.0 °C were the highest and became saturated in the presence of either SET1 and SET2 (Figure 2). The same experiments were performed using 1.1 μL of SD6.0 and SD8.0 as templates, with similar results obtained (data not shown). These results indicated that annealing/extension reactions ranging from 57.0 °C to 62.4 °C were optimal.

### 3.6. Quantitative Evaluation Using Samples Extracted from Plasma and Urine

We extracted p.horseEPO from 1.01 mL of plasma collected in EDTA collection tubes and 1.01 mL of urine (each spiked with 10 μL of SD), followed by quantification using 8.8 μL of the extracts to increase sensitivity.

**Figure 3:**
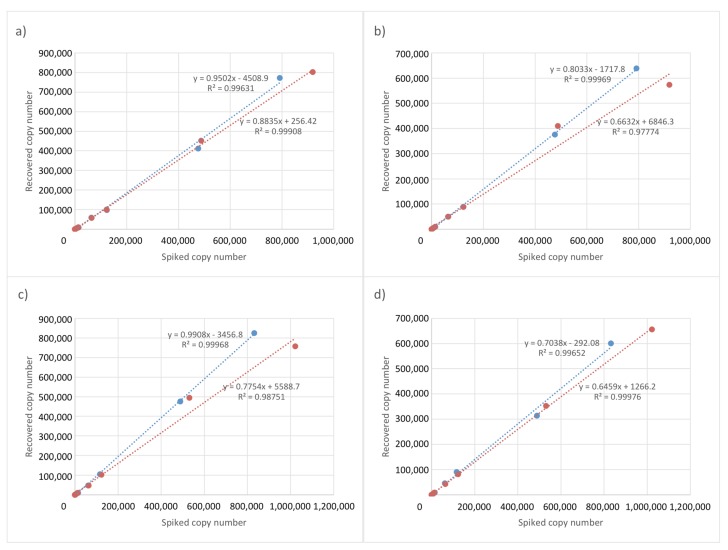
Efficient and quantitative recovery using samples extracted from plasma and urine. (**a**) Plasma and (**b**) urine samples in EDTA collection tubes at day 1 and (**c**) plasma and (**d**) urine samples at day 2. Blue circles denote SET1, and red circles denote SET2. The horizontal axis shows the spiked copy number in 1 mL of plasma and urine, respectively, and the vertical axis shows copy numbers recovered from plasma and urine, respectively. The slope indicates the recovery rate.

Recovery rates from the spike-recovery test using extracts from plasma in EDTA collection tubes resulted in correlation coefficients of 0.9963 and 0.9991 for SET1 and SET2 for Spiked_SD4.0 through Spiked_SD8.5 at day 1, respectively, with extraction efficiencies of 0.9502 and 0.8835, respectively (Figure 3a), and minimum recovery rates of 76.2% and 66.9%, respectively (Table 3). For day 2, the correlation coefficients were 0.9997 and 0.9875 for SET1 and SET2 using Spiked_SD4.0 through Spiked_SD8.5, respectively, with extraction efficiencies of 0.9908 and 0.7754, respectively (Figure 3c), and minimum recovery rates of 72.5% and 63.1%, respectively (Table 3).

The recovery rates from urine showed correlation coefficients of 0.9997 and 0.9777 for SET1 and SET2 using Spiked_SD4.0 through Spiked_SD8.5 at day 1, respectively, with extraction efficiencies of 0.8033 and 0.6632, respectively (Figure 3b) and minimum recovery rates of 71.9% and 50.4%, respectively (Table 3). For day 2, the correlation coefficients were 0.9965 and 0.9998 for SET1 and SET2 using Spiked_SD4.0 through Spiked_SD8.5, respectively, with extraction efficiencies of 0.7038 and 0.6459, respectively (Figure 3d), and minimum recovery rates of 63.2% and 40.6%, respectively (Table 3).

These results indicated that recovery rates from plasma and urine were ~60% and ~40%, respectively. Compared with plasma extracts, we observed slightly lower ddPCR amplification efficiency for urine extracts, likely due to the incomplete removal of PCR inhibitors in the urine. These results demonstrated successful extraction and quantification of the *EPO* transgene in the presence of at least 130 and 200 copies in 1 mL of plasma and urine, respectively, which is equivalent to 0.87 and 0.89 copies/μL of DNA extracts (recovery rate: 60% and 40%, respectively; extract volume: 90 μL). Use of 8.8 μL of template resulted in *EPO* transgene copy number at >7 copies for the ddPCR reaction (20 μL), which was the minimum necessary for quantification.

### 3.7. Casework Example

Gene-doping detection of the horse *EPO* transgene was performed using 8.8 μL of extracts from plasma and urine obtained from 46 thoroughbred racehorses. ddPCR products amplified by both SET1 and SET2 were not observed in either plasma or urine; however, some samples showed amplification of very low copy number in the presence of either SET1 (0.04 ± 0.10 copies for plasma and 0.09 ± 0.12 copies for urine) or SET2 (0.02 ± 0.05 copies for plasma and 0.02 ± 0.06 copies for urine). This is likely due to nonspecific amplification, because the racehorses sampled here have not been administered the *EPO* transgene for any treatments. These results indicated that both SET1 and SET2 were necessary to protect against false positive results.

Notably, it would be difficult to completely prevent nonspecific amplification based on PCR theoretically amplifying up to 10^9^ copies across 30 cycles. Therefore, it would be more appropriate to define a detection threshold. Based on our results, 7 copies per reaction tube (20 μL) would be a suitable threshold.

### 3.8. Detection of pBApo.horseEPO After Administration to a Horse

**Figure 4:**
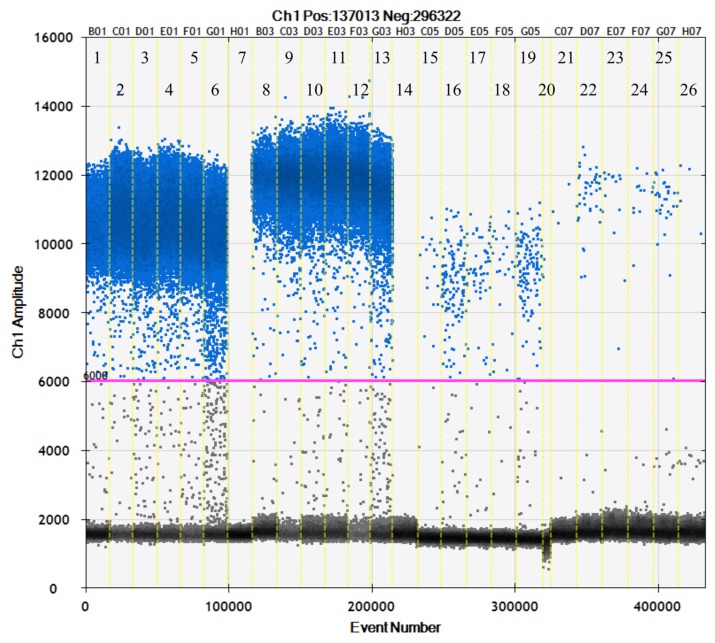
ddPCR detection of the *EPO* transgene in plasma and urine after intramuscular administration to a horse. The horizontal axis shows the number of each sample. Times are all post-administration. Plasma: lanes 1 and 8: 15 min; lanes 2 and 9: 1 h; lanes 3 and 10: 3 h; lanes 4 and 11: 6 h; lanes 5 and 12: 12 h; lanes 6 and 13: 1 day; and lanes 7 and 14: 2 days. Urine: lanes 15 and 21: 50 min; lanes 16 and 22: 3 h; lanes 17 and 23: 6 h; lanes 18 and 24: 12 h; lanes 19 and 25: 1 day; and lanes 20 and 26: 2 days. Lanes 1 through 7 and 15 through 20 were amplified using SET1, and lanes 8 through 14 and 21 through 26 were amplified using SET2. The vertical axis shows PCR amplitudes (fluorescence intensity). The pink line is the detection threshold. ddPCR amplification of lanes 1 through 14 was performed using SDs of DNA extracts; therefore, amplification was not observed for 2-day samples (lanes 7 and 14).

**Figure 5:**
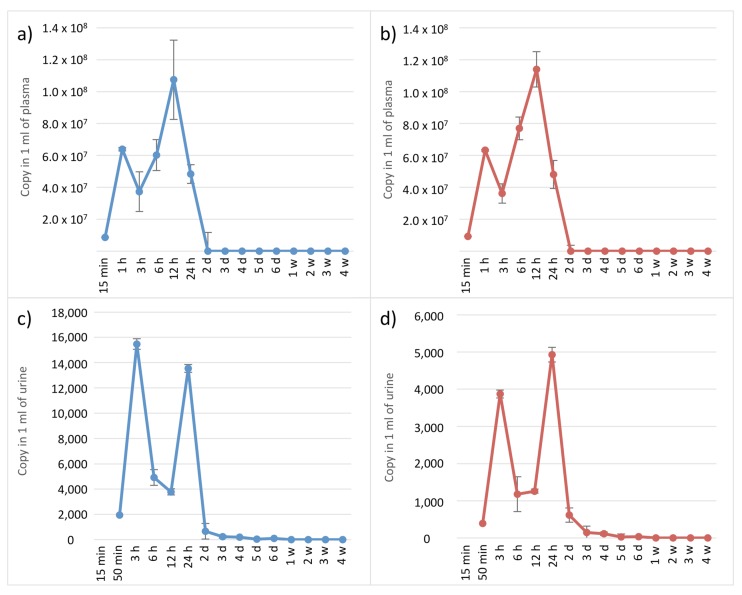
Copy number of the *EPO* transgene detected in 1 mL of plasma and urine, respectively, following plasmid administration to a horse. (**a**) Copy number amplified by SET1 using extracts from plasma, (**b**) copy number amplified by SET2 using extracts from plasma, (**c**) copy number amplified by SET1 using extracts from urine, (**d**) copy number amplified by SET2 using extracts from urine.

Figure 4 and Figure 5 show the detection results from 1 mL of plasma and urine, respectively, collected in EDTA collection tubes from a thoroughbred horse intramuscularly injected with 20 mg of pBApo.horseEPO. At time points ranging from 15-min to 1-day post-administration, large copy numbers of the *EPO* transgene were detected using both SET1 and SET2 from plasma. Interestingly, a bimodal peak was observed for samples collected at 1-h and 12-h post-administration, suggesting that some plasmids might have remained at the administration site. Although a small number of *EPO* transgene copies was detected at 2-days post-administration, ddPCR amplification was not detected from samples collected 3-days to 4-weeks post-administration (Figure 5, Table S1). Samples from plasma collected in heparin collection tubes showed poor amplification (data not shown).

We detected a small number of *EPO* transgene copies from urine samples collected from 15-min to 2-days post-administration using both SET1 and SET2 (Figure 4 and Figure 5), similar to results for plasma samples, although the detection peaks differed between urine and plasma samples. Because urine accumulates in the bladder, peak *EPO* content in urine might occur at a later time point relative to that in plasma. Immediately after intramuscular injection, a large number of *EPO* copies were present in circulating blood, followed by degradation within 1- to 2-days post-administration and/or excretion in urine. It was indicated that almost all the transgenes were degraded within 1 or 2 days. These results suggest that this method was capable of detecting *EPO* from plasmid DNA within days following intramuscular injection.

In our previous study involving intramuscular injection of plasmid DNA into a microminipig [16], we observed large-scale degradation of the plasmid within 1 day of administration, followed by sustainable detection for up to 3 weeks. This difference in detection results is likely a consequence of metabolic or size differences between animals.

No changes were observed in red blood-cell count or *EPO* concentration before and after plasmid administration (data not shown). The dose used in this experiment was four-fold greater than that used in a previous study [10], but because it was administered via a naked plasmid, it was likely that the concentration of the *EPO* transgene was inadequate to allow uptake into cells. This was supported by the large number of plasmids detected in blood samples taken immediately after administration. For plasmid-based gene therapy, administration using gold nanoparticles might be necessary [17].

## 4. Conclusions

Conventional real-time PCR provides quantitative information through the use of a standard calibration curve, whereas digital PCR allows absolute quantitation to determine the copy number of the targeted sequences without a standard calibration curve. Moreover, ddPCR has a further advantage in that it is unaffected by amplification efficiency, which is dependent upon the PCR conditions. Therefore, our findings suggest ddPCR as a suitable technology for the detection of gene-doping.

In this study, we demonstrated the detection of an *EPO* transgene in thoroughbred racehorses using the following procedures: (1) blood sampling into an EDTA collection tube, (2) plasma separation by centrifugation, (3) DNA extraction from 1 mL of plasma and/or urine using magnetic beads, and (4) ddPCR detection using 8.8 μL of the respective extract. These results demonstrated the efficacy of this method to detect at least 130 and 200 copies of the *EPO* transgene in 1 mL plasma and urine, respectively. These procedures might be applicable for other transgenes, such as *insulin-like growth factor 1* and *follistatin*, which are also potential targets for gene-doping in racehorses [4].

## Figures and Tables

**Table 1:** Primers and probes used for ddPCR.

Marker	Primer/Probe Name	Sequence	Sequence Position
SET1 *	Forward primer_eX	XXXXXXXXXXXXXXXXXXX	exon X
	Reverse primer_eX	XXXXXXXXXXXXXXXXXXXXXX	exon X
	FAM_TaqMan MGB Probe_X/X	XXXXXXXXXXXXXXXXXX	exon junction X/X
SET2	Forward primer_e2	CAGCCTCACCTCCCTGCTT	exon 2
	Reverse primer_e3	CACAAAGTATCAACAGCGAATGTTC	exon 3
	FAM_TaqMan MGB Probe_2/3	AGCCCAGAAGGAAG	exon junction 2/3

* Sequence information for SET1 is withheld to support the integrity of gene-doping tests.

**Table 2:** Concentrations of reference material (RM) and serially diluted (SD) samples.

	Mass Concentration (ng/μL) *	Theoretical Copy Number Concentration (Copy/μL) **	**Day 1**			
			**SET1**		**SET2**	
			**Measured Copy Number Concentration by ddPCR (Copy/μL)**	**Measured/Theoretical Ratio**	**Measured Copy Number Concentration by ddPCR (Copy/μL)**	**Measured/Theoretical Ratio**
RM	8.01	1.76 × 10^9^	-	-	-	-
SD1.0	-	1.76 × 10^8^	-	-	-	-
SD2.0	-	1.76 × 10^7^	-	-	-	-
SD3.0	-	1.76 × 10^6^	-	-	-	-
SD4.0	-	1.76 × 10^5^	7.92 × 10^4^ ± 2.96 × 10^4^	0.450	9.19 × 10^4^ ± 2.96 × 10^4^	0.522
SD4.5	-	8.81 × 10^4^	4.76 × 10^4^ ± 8.93 × 10^3^	0.540	4.88 × 10^4^ ± 1.56 × 10^4^	0.554
SD5.0	-	1.76 × 10^4^	1.23 × 10^4^ ± 3.07 × 10^3^	0.699	1.22 × 10^4^ ± 2.00 × 10^3^	0.693
SD5.5	-	8.81 × 10^3^	6.35 × 10^3^ ± 8.00 × 10^2^	0.721	6.39 × 10^3^ ± 1.48 × 10^3^	0.725
SD6.0	-	1.76 × 10^3^	1.30 × 10^3^ ± 73	0.739	1.41 × 10^3^ ± 1.28 × 10^2^	0.801
SD6.5	-	8.81 × 10^2^	7.27 × 10^2^ ± 1.61 × 10^2^	0.825	6.01 × 10^2^ ± 41	0.682
SD7.0	-	1.76 × 10^2^	1.19 × 10^2^ ± 6.4	0.676	1.18 × 10^2^ ± 7.6	0.670
SD7.5	-	88.1	57.3 ± 8.3	0.650	66.7 ± 15	0.757
SD8.0	-	17.7	13.3 ± 4.7	0.751	12.8 ± 0.2	0.723
SD8.5	-	8.8	6.3 ± 4.2	0.716	5.8 ± 1.9	0.659
SD9.0	-	1.8	0.5 ± 0.9	0.278	0.5 ± 0.8	0.278
SD9.5		0.9	2.0 ± 2.4	2.222	0	0
	Mass Concentration (ng/μL) *	Theoretical Copy Number Concentration (Copy/μL) **	**Day 2**			
			**SET1**		**SET2**	
			**Measured Copy Number Concentration by ddPCR (Copy/μL)**	**Measured/Theoretical Ratio**	**Measured Copy Number Concentration by ddPCR (Copy/μL)**	**Measured/Theoretical Ratio**
RM	8.01	1.76 × 10^9^	-	-	-	-
SD1.0	-	1.76 × 10^8^	-	-	-	-
SD2.0	-	1.76 × 10^7^	-	-	-	-
SD3.0	-	1.76 × 10^6^	-	-	-	-
SD4.0	-	1.76 × 10^5^	8.31 × 10^4^ ± 2.98 × 10^4^	0.472	1.02 × 10^5^ ± 2.61 × 10^4^	0.580
SD4.5	-	8.81 × 10^4^	4.89 × 10^4^ ± 1.19 × 10^4^	0.555	5.30 × 10^4^ ± 9.20 × 10^3^	0.602
SD5.0	-	1.76 × 10^4^	1.16 × 10^4^ ± 7.41 × 10^2^	0.659	1.23 × 10^4^ ± 1.02 × 10^3^	0.699
SD5.5	-	8.81 × 10^3^	6.12 × 10^3^ ± 6.95 × 10^2^	0.695	6.35 × 10^3^ ± 3.52 × 10^2^	0.721
SD6.0	-	1.76 × 10^3^	1.39 × 10^3^ ± 1.65 × 10^2^	0.790	1.18 × 10^3^ ± 1.85 × 10^2^	0.670
SD6.5	-	8.81 × 10^2^	6.41 × 10^2^ ± 6.4	0.728	6.45 × 10^2^ ± 1.63 × 10^2^	0.732
SD7.0	-	1.76 × 10^2^	94.0 ± 12.2	0.534	1.14 × 10^2^ ± 22.5	0.648
SD7.5	-	88.1	60.0 ± 6.9	0.681	65.3 ± 2.3	0.741
SD8.0	-	17.7	6.8 ± 1.9	0.384	15.1 ± 6.8	0.853
SD8.5	-	8.8	3.8 ± 3.6	0.432	4.8 ± 3.3	0.545
SD9.0	-	1.8	2.0 ± 2.1	1.111	1.4 ± 1.3	0.778
SD9.5		0.9	0.4 ± 0.7	0.444	0.5 ± 0.8	0.556

* Quantified range: 0.2–100 ng/μL. ** Theoretical copy number concentration was calculated from the RM weight concentration, and SD samples were converted from the RM based on the dilution. Samples were converted from the RM based on the serial dilution.

**Table 3:** Extraction efficiency of spiked plasmid DNA from EDTA collection tubes and urine.

**Day 1**								
**Spiked SD in 1 mL of Plasma Or Urine**	**Plasma**				**Urine**			
	**SET1**		**SET2**		**SET1**		**SET2**	
	**Recovered Conc.**	**Recovery Rate**	**Recovered Conc.**	**Recovery Rate**	**Recovered Conc.**	**Recovery Rate**	**Recovered Conc.**	**Recovery Rate**
Spiked_SD4.0	8575 ± 1429	97.4%	8908 ± 1518	87.3%	7100 ± 463	80.7%	6375 ± 130	62.5%
Spiked_SD4.5	4579 ± 386	86.5%	5026 ± 44	92.7%	4173 ± 518	78.8%	4559 ± 235	84.1%
Spiked_SD5.0	1088 ± 16	79.4%	1118 ± 46	82.5%	990 ± 13	72.2%	982 ± 29	72.5%
Spiked_SD5.5	660 ± 32	93.6%	646 ± 47	91.0%	553 ± 22	78.3%	558 ± 22	78.6%
Spiked_SD6.0	115 ± 7.8	80.0%	105 ± 5.7	66.9%	104 ± 1.3	71.9%	111 ± 5.9	71.0%
Spiked_SD6.5	62 ± 2.1	76.2%	57 ± 0.7	85.0%	60 ± 3.7	74.0%	59 ± 2.5	88.4%
Spiked_SD7.0	11 ± 2.6	85.3%	12 ± 1.4	90.4%	11 ± 2.3	81.5%	9.6 ± 1.0	72.7%
Spiked_SD7.5	6.3 ± 1.1	99.4%	5.6 ± 0.6	75.4%	4.7 ± 0.4	73.3%	5.4 ± 0.5	73.1%
Spiked_SD8.0	1.6 ± 0.5	108.6%	1.4 ± 0.5	97.3%	1.2 ± 0.6	81.6%	0.7 ± 0.3	50.4%
Spiked_SD8.5	0.7 ± 0.6	93.6%	0.7 ± 0.2	102.2%	0.6 ± 0.5	91.2%	0.5 ± 0.1	78.9%
**Day 2**								
**Spiked SD in 1 mL of Plasma Or Urine**	**Plasma**				**Urine**			
	**SET1**		**SET2**		**SET1**		**SET2**	
	**Recovered Conc.**	**Recovery Rate**	**Recovered Conc.**	**Recovery Rate**	**Recovered Conc.**	**Recovery Rate**	**Recovered Conc.**	**Recovery Rate**
Spiked_SD4.0	9167 ± 989	99.2%	8417 ± 792	74.2%	6675 ± 218	72.3%	7292 ± 823	64.3%
Spiked_SD4.5	5279 ± 539	97.2%	5494 ± 251	93.2%	3488 ± 540	64.2%	3915 ± 72	66.4%
Spiked_SD5.0	1174 ± 59	90.9%	1146 ± 37	83.9%	1008 ± 44	78.0%	911 ± 10	66.7%
Spiked_SD5.5	535 ± 24	78.7%	532 ± 23	75.4%	513 ± 35	75.5%	480 ± 33	68.1%
Spiked_SD6.0	113 ± 2.9	73.1%	108 ± 12	82.9%	98 ± 3.0	63.2%	97 ± 7.0	74.2%
Spiked_SD6.5	57 ± 2.8	79.8%	56 ± 1.5	77.0%	51 ± 4.1	70.9%	49 ± 7.0	67.9%
Spiked_SD7.0	12 ± 1.7	118.1%	12 ± 0.5	91.6%	10 ± 1.4	98.9%	9.6 ± 2.5	75.2%
Spiked_SD7.5	4.8 ± 0.6	72.5%	4.6 ± 0.3	63.1%	5.0 ± 0.5	75.0%	4.6 ± 1.5	63.1%
Spiked_SD8.0	0.8 ± 0.7	102.7%	1.4 ± 0.3	82.6%	1.6 ± 0.9	211.9%	0.8 ± 0.6	49.8%
Spiked_SD8.5	0.5 ± 0.3	118.4%	0.5 ± 0.1	89.1%	0.8 ± 0.3	195.4%	0.2 ± 0.4	40.6%

Recovered conc.: Copy number concentration (copy/μL) of DNA soutions extracted from 1.01 mL of spiked samples. Recovered rate (%) = (Recovered conc. × 90 μL of elution buffer)/(10 μL × Spiked_SD conc.) × 100.

## Data Availability

Primer and probe sequence information of SET1 is masked for gene-doping control. Sequence information will be provided by a confidentiality agreement with the corresponding author.

## Supplementary Materials

The following is available online at https://www.mdpi.com/2073-4425/10/3/243/s1: Table S1: Copy number of the *EPO* transgene detected in 1 mL of plasma and urine, respectively, after intramuscular administration to a horse.
