# Supplementary material for: Droplet Digital PCR Detection of the Erythropoietin Transgene from Horse Plasma and Urine for Gene-Doping Control
Source: Genes (Basel). 2019 Mar 21;10(3):243. doi: 10.3390/genes10030243 (PMC6471249; doi:10.3390/genes10030243)
Supplement: Supplementary file 1 [file genes-10-00243-s001.pdf]

Table S1. Copy number of the EPO transgene detected in 1 mL of plasma and urine, respectively, after intramuscular administration to a horse.

Collection time	Plasma		Urine			
	SET1	SET2	Sampled volume (ml)	pH	SET1	SET2
15 min	8.69E+06	9.30E+06	-	-	-	-
50 min	-	-	800	6.7	1943	390
1 hour	6.39E+07	6.32E+07	-	-	-	-
3 hours	3.73E+07	3.62E+07	800	6.7	15465	3870
6 hours	6.02E+07	7.70E+07	800	6.4	4920	1178
12 hours	1.07E+08	1.14E+08	1000	6.2	3780	1260
1 day	4.84E+07	4.81E+07	800	7.2	13545	4928
2 days	1327.5	1147.5	800	6.8	668	615
3 days	< LOD	< LOD	1000	7.1	233	147
4 days	nd	< LOD	700	7.2	196	116
5 days	< LOD	< LOD	1000	7.4	< LOD	< LOD
6 days	< LOD	< LOD	400	7.5	107	< LOD
1 week	< LOD	nd	800	7.5	nd	nd
2 weeks	154	79	1000	7.2	nd	nd
3 weeks	nd	< LOD	800	7.0	nd	nd
4 weeks	< LOD	nd	500	7.2	nd	nd

nd: not detected

< LOD: under Limit of Detection in this study
